# PromoterCAD: data-driven design of plant regulatory DNA

**DOI:** 10.1093/nar/gkt518

**Published:** 2013-06-12

**Authors:** Robert Sidney Cox, Koro Nishikata, Sayoko Shimoyama, Yuko Yoshida, Minami Matsui, Yuko Makita, Tetsuro Toyoda

**Affiliations:** ^1^Bioinformatics and Systems Engineering Division (BASE), RIKEN, 1-7-22 Suehiro-cho, Tsurumi-ku, Yokohama, Kanagawa 230-0045, Japan, ^2^Integrated Database Unit, Advanced Center for Computing and Communication (ACCC), RIKEN, 2-1 Hirosawa, Wako, Saitama 351-0198, Japan and ^3^Synthetic Genomics Research Team, Biomass Engineering Program (BMEP), RIKEN, 1-7-22 Suehiro-cho, Tsurumi-ku, Yokohama, Kanagawa 230-0045, Japan

## Abstract

Synthetic promoters can control the timing, location and amount of gene expression for any organism. PromoterCAD is a web application for designing synthetic promoters with altered transcriptional regulation. We use a data-first approach, using published high-throughput expression and motif data from for *Arabidopsis thaliana* to guide DNA design. We demonstrate data mining tools for finding motifs related to circadian oscillations and tissue-specific expression patterns. PromoterCAD is built on the LinkData open platform for data publication and rapid web application development, allowing new data to be easily added, and the source code modified to add new functionality. PromoterCAD URL: http://promotercad.org. LinkData URL: http://linkdata.org.

## INTRODUCTION

Promoter sequences are collections of *cis*-regulatory motifs that determine interactions between transcription factors and the basal transcriptional apparatus. There are several methods for recognizing *cis*-regulatory motifs within promoter sequences: forming a position weight matrix from experimentally confirmed binding sites (1–4), word frequency analysis of short sequences at each promoter position (5) and correlation of motif presence with similar expression profiles (6). These motif recognition methods can also be used to make functional predictions of new combinations of *cis*-regulatory motifs with basal sequences: synthetic promoters.

Recent software tools have been developed specifically for DNA design, including GenoCAD (7), Eugene (8), DeviceEditor (9), J5 (10), TinkerCell (11), Genome Compiler and Gene Designer (12). Although these tools have many uses for designing genes, proteins and pathways, they do not work at the *cis-*regulatory motif level and are not directly useful for arranging motifs into synthetic regulatory promoter sequences. Tools such as Synbioss Designer (13) do allow for the design of combinatorial bacterial promoters (14), but the source data used are currently limited to the BioBricks parts registry (15). To our knowledge, there are no tools specifically for promoter design.

### Plant promoters have complex motif patterns

Plants have many transcription factors, and *Arabidopsis thaliana* has a compact genome: resulting in promoters with dense clusters of *cis-*regulatory motifs ∼500 bp upstream of the transcription start site. The *Cauliflower Mosaic Virus 35S promoter* (*CaMV35S*) has been shown to express at high levels across many tissues in dicotyleodon flowering plants. Specific regulatory domains of this promoter correspond to expression in tissue organs, such as roots, leaves and flowers (16), and synthetic promoters based on *CaMV35S* have been used to identify the functions of the individual *cis*-regulatory motifs within 400 bp of the transcriptional start site (17). These properties have made *CaMV35S* a model multicellular eukaryotic promoter for high *cis-*regulation density and a platform for the design of synthetic promoters in plants.

The minimal promoter region, ∼45 bp upstream of the transcriptional start site, is necessary but not sufficient for *CaMV35S* expression (18). This region includes important sequences for strongly regulated TATA-type promoters (5): the TATA box, a plant-specific CT-rich region called the Y-patch, and the initiator region surrounding the transcription start site. Many chimeric promoters fuse the CaMV35S minimal promoter region to regulatory upstream sequences (17). Typically, the natural promoter of interest is aligned to *CaMV35S* (aligning either the transcriptional start site or a TATA box), with the upstream sequence coming from the regulated promoter and the minimal promoter region from *CaMV35S*. This minimal promoter can be used as a starting point for exploring the effects of synthetically introduced *cis-*regulatory motifs.

### Synthetic plant promoter design by motif arrangement

**Figure 1.**
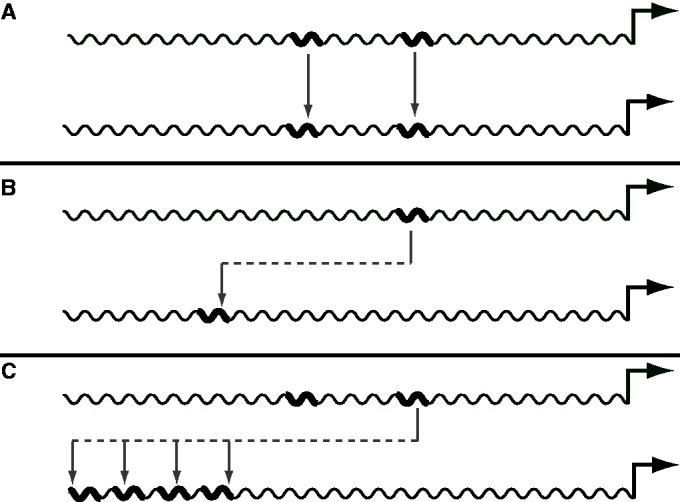
Motif editing and placement. Natural *cis*-regulatory motifs can be placed into a synthetic promoter sequence in several ways. The motifs can be copied into the corresponding position of the synthetic promoter sequence (**A**), or genome sequence analysis can be used to predict functional locations of a motif (**B**). Either of these two techniques can be combined with a strategy of stacking multiple copies of a motif (**C**), which helps to ensure that at least one copy is in a functional location. The default functionality of PromoterCAD is (A), and (B) and (C) are optional and can be specified in the advanced motif view.

Position-specific motifs can be added to a synthetic promoter at their natural location, by prediction of their maximally effective location (19), or by stacking of multiple copies of *cis*-regulatory motifs upstream of a chosen site (20) (Figure 1). These placement strategies have been used to create functionally equivalent but sequence divergent synthetic versions of *CaMV35S*, to reduce homology-dependent transcriptional silencing. One study moved the *as-1* motif and TATA box to the corresponding location a synthetic random sequence and showed that the CaMV35S expression level could be maintained (18). Another study showed that three neighboring motifs cooperatively confer a salt stress response (1). These motif operations have been experimentally shown to permit rational design of synthetic arrangements of *cis*-regulatory motifs in plants.

### PromoterCAD

We wish to create functional and modular sequences (‘CAD bricks’) for the design of synthetic genomes (21). Such design elements can be culled from published genomic and expression databases. To empower the process of synthetic promoter design, we built the PromoterCAD web server, an open set of tools for mining gene expression and *cis*-regulatory motif data, and arranging retrieved motifs. PromoterCAD allows collections of motifs to be added to natural or synthetic baseline promoter sequences. PromoterCAD is intended to be used by *Arabidopsis* researchers to enable the design of synthetic promoter sequences and as a data mining tool for uncovering genes with useful expression patterns.

The PromoterCAD tools MotifExpress and MotifCircadian (described in ‘Results’ section) allow for data mining of candidate functional sequences. These tools use high-throughput gene expression data (e.g. microarrays) to locate genes with useful expression patterns. Then, a regulatory motif recognition method is chosen, and motifs from the chosen gene are displayed in alignment with the sequence design. The user adds one or more motifs to the design, using the PromoterCAD UI to arrange them. Finally, a ‘baseline’ sequence can be used to fill in the remaining sequence, either taken from a natural promoter or specified by the user.

Using a simple menu-driven UI, PromoterCAD incorporates rich databases and flexible user choices. At each step of the design process, PromoterCAD provides links to data files, visualizations and additional explanations and references to instruct the user. PromoterCAD tracks each choice the user makes and each result in the workflow history that can be saved as a public or private snapshot on LinkData.org. This creates a sequence design submission function to allow for design publication, collaboration and checking. The web application and linked data hosting system at LinkData.org also allows easy user modification of PromoterCAD, to add new data sets and functions.

## MATERIALS AND METHODS

### Data collection

We collated previously published genomic and transcriptomic data, including information on 21 000 genes from *A. thaliana* and 1 410 000 microarray data measurements in 20 growth conditions and 79 tissue organs and developmental stages. *AtGenExpress* (22) Developmental *A.thaliana* gene expression measurements: AtGenExpress is a microarray database that includes measurements from the main plant tissue organs and growth stages. *DIURNAL* (23) Circadian *A. thaliana* microarray measurements: the DIURNAL project is a microarray gene expression database that was collected >2 days (44 h) at 4-h intervals in various nutrient, light and temperature growth conditions. These measurements are made on 7- to 9-day-old seedlings and show gene expression levels across the whole plant. *ATTED-II* (19) database: uses gene co-expression analysis of the AtGenExpress data to predict 304 7-bp motifs in promoters within a promoter region 200 bp upstream of the transcription start site. *PPDB* (24) The Plant Promoter Database: uses word frequency analysis to identify 308 8-bp motifs within a promoter region 500 bp upstream of the transcription start site. We used the gene name [TAIR Gene Locus ID (25)] to align the gene expression values with the motif locations (Supplementary Figure S1).

### Data processing

Each data collection and processing step was recorded and can be checked online (Supplementary Table S1 and Supplementary Figure S2). The raw motif data were collected as the distance between the center base pair of a motif and the transcription start site (26). Gene expression data from AtGenExpress presented as triplicate log-scale robust multiarray analysis [RMA (27)]. From the triplicate data, the user can choose either the mean or the median for data mining with MotifExpress. For the DIURNAL data set, the data were presented as the RMA exponential base 2. We fit the 12 measurement points to a sine function using the *nlinfit* function of MATLAB (The Mathworks, Supplementary Figure S3). Each gene expression data property (absolute expression, normalized expression, circadian amplitude and so forth) was sorted to create lists of genes with the highest to lowest values of that property. These rank lists are used as inputs to the gene expression mining tools and allow for an online user interface—all functions can be executed in the browser without the need for job submission.

### Web server application development

PromoterCAD is designed with a modular code structure where the gene expression mining tools are loadable plugins. PromoterCAD written in Javascript, and built on the data and web application development platform LinkData.org (Supplementary Information). Data visualization plots use the non-commercial HighCharts JS library (highsoft.com). The source code of PromoterCAD is licensed under the LGPL-3.0 license (the GNU Lesser General Public License, version 3.0) and the Creative Commons license CC-BY-SA, version 3.0. For LinkData applications, such as PromoterCAD, we recommend browsers Firefox 12 or later and Google Chrome 19 or later.

## RESULTS

### Gene expression mining tools

*MotifExpress* finds the gene with the corresponding highest/lowest expression level in a specific tissue or time of day (for additional description see Supplementary Figure S4) and returns motifs identified from that gene. The user selects a gene expression property (such as the expression level in a particular plant tissue or time of day) and whether a maximum or minimum value is desired. When acting on a normalized property, MotifExpress will return the gene that has the highest or lowest expression in a given condition relative to is mean expression level—this can be used to identify genes that are highly specific to a tissue or time of day. In cases where no motifs are found in the region, the next most extreme gene is used. MotifExpress was modified by PromoterCAD user Masahiro Mochizuki to create a new tool: MotifRanking. The MotifRanking tool allows selection from 10 variant genes for further customization of the gene expression pattern.

*MotifCircadian* finds genes with the largest circadian amplitudes (for additional description see Supplementary Figure S4). The user selects the growth condition and motif data set, and then chooses the growth phase of the gene expression profile, corresponding to the time of day when the expression is highest. When acting on normalized data, MotifCircadian finds the gene with the largest normalized amplitude (fold-change).

*InputMotif* allows motifs to be added from the literature or other software. As users may have previous knowledge of specific regulatory motifs, we provide a simple interface to allow manually input motifs. The user can paste or type motif sequence text and position (from the motif center base pair relative to the transcription start site). The motif is aligned along the promoter design sequence; therefore, the user can inspect the position and decide which motifs to include. The motif can be moved, repeated, or deleted by using the advanced motif view, as described later in the text.

### Motif overlap conflict resolution

PromoterCAD provides a system for collecting many motifs into a promoter design, and to arbitrate when they overlap. The default motif placement is the same location as in the natural promoter, relative to the transcription start site. This operation can be used to add a single motif, or to collect all annotated motifs from the natural promoter. When an introduced motif overlaps with a previously added motif, the user is asked with a dialog box to decide whether the old or new motif sequence should be used in the overlapping region. After each motif operation, altered base pairs are highlighted in red. This system allows sets of motifs to be placed simultaneously and resolves when such placements create conflicts.

### Advanced motif editing view

**Figure 2.**
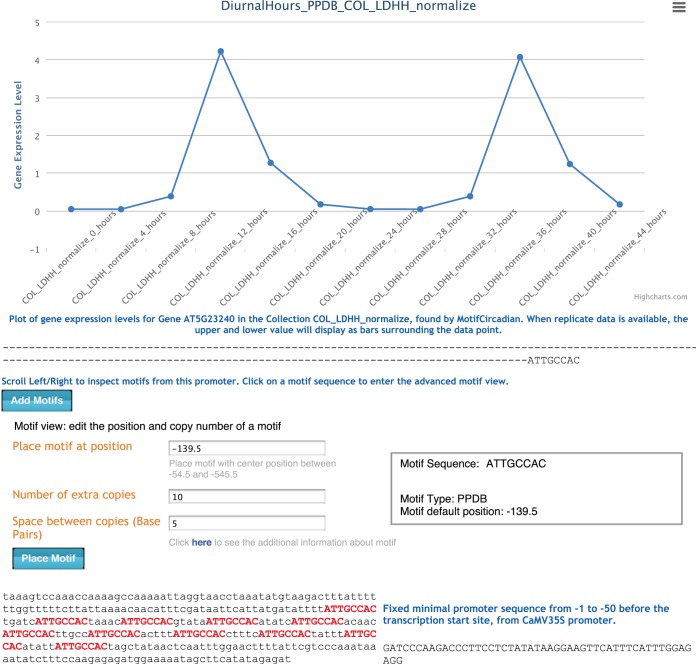
Web interface system of PromoterCAD guides DNA design. Here the MotifCircadian tool returns the strongly circadian gene AT5G23240 with a maximum phase of 8 hours. For each menu choice, tooltips explain the details of methods and experiments. The expression data of the gene is plotted over the data category: the circadian gene expression level over two days of plant growth. The motif ATTGCCAC identified by the chosen motif analysis method PPDB is presented as an alignment with the blank promoter design. Clicking on the motif sequence reveals the motif editing view, where 10 extra upstream copies of the motif are placed with a user defined spacing of 5 base-pairs. This set of 10 motifs in the design is placed into the ‘background’ sequence from the natural promoter AT5G23240.

Clicking on a motif aligned with the promoter sequence brings up an advanced motif view. PromoterCAD allows the user to directly specify the motif placement by the middle base pair position of the motif (for motifs with an even number of base pairs this position is a half integer). In addition to the position, a suggested location is provided by default, based on a guess from the motif data set (Supplementary Information). The user can add multiple copies of a motif with a specified interval of base pairs in between each copy (Figure 2).

### Gene expression mining output and visualizations

The PromoterCAD tools return gene and motif information, including gene expression plots, motif alignments and external links. For AtGenExpress data, this plots the gene expression level in similar tissues (such as Flowering, Leaf, Root and so forth), with the triplicate data plotted to show experimental reliability (Figure 2). For the DIURNAL data set, the expression profile is plotted over 48 h. External links supply additional information and data visualization regarding the gene locus in popup windows. These links include the *Arabidopsis* Information Resource page for the gene locus (25), the PromoterCAD data files on LinkData.org and the ATTED-II and PPDB motif analysis web server pages. Links to the eFP browser (28) and the HanaDB tissue visualization (29) provide images of the different *A. thaliana* developmental tissues with the gene expression level shown as a color scale (Figure 2). These links and external visualizations inform the user to decide which motifs to incorporate into the design.

### Sequence output data

The output of PromoterCAD is a promoter sequence and the series of operations used to construct it from the baseline sequence. Each design step records the data sources used, Gene Locus ID, PromoterCAD tool, gene expression data property, motifs found and the motif positions. In general, a promoter design will consist of a sequence of motifs separated by empty base pairs (represented as dashes). Empty base pairs can be filled in using the natural promoter sequence at the final design step.

### LinkData system for data sharing, collaboration and rapid application customization

PromoterCAD is built on LinkData (linkdata.org), a rapid web development and semantic data system that combines a data repository and application repository, including accessibility controls for both source code and data. LinkData allows users record and publish PromoterCAD DNA designs, to customize PromoterCAD by adding new data and to extend the functionality through code forking. These features allow PromoterCAD to become an online collaborative design tool, as different users can easily replicate and check others’ workflows.

## DISCUSSION

Synthetic promoters will be critical components for controlling introduced genes and metabolic pathways in designed synthetic genomes. PromoterCAD facilitates rapid design of functional regulatory DNA sequences from genomic and expression data. Mashups of *cis*-regulatory motif analysis databases (ATTED-II, PPDB) with gene expression databases (AtGenExpress, DIURNAL) allow the user to perform advanced data mining operations from a simple menu interface. The data sources are described with tooltips inside the program (along with detailed external references), with links referencing original data sources and publications, allowing non-experts to use PromoterCAD to design candidate synthetic promoters with a fast learning cycle. PromoterCAD gene expression mining tools search for gene expression properties: such as gene expression level in particular plant tissues (MotifExpress), or phase and amplitude of circadian oscillations (MotifCircadian). PromoterCAD integrates these tools with flexible DNA sequence editing operations.

Biologists and DNA designers familiar with particular *cis*-regulatory motifs can easily add them with InputMotif. Users can upload additional gene expression and motif data using the LinkData upload and input data system. Current data sources can be re-processed, such as by using different normalization procedures. This also allows researchers to check the data pre-processing steps used to prepare source data for PromoterCAD. By following the data pre-processing stages on the LinkData work pages (Supplementary Table S1), users can add entirely new data and functionality.

PromoterCAD uses the LinkData interconnected data repository and code repository. The PromoterCAD source code can be easily forked, allowing programmers and researchers to use the rapid development environment to create new expression mining tools from the data sources. The modular structure of the PromoterCAD code could allow the extension to new organisms. Using similarly structured data, only the natural promoter sequences and links to external information need to be changed.

PromoterCAD was used for GenoCon2, the international rational genomic design contest (genocon.org), where contestants used PromoterCAD to design synthetic plant promoters for specific tissue and time of day expression in *A. thaliana*. Forty promoter designs were submitted to the contest, and 10 designs have been chosen for experimental characterization. These synthetic promoters will be synthesized, transformed into plants and measured for their temporal expression pattern using a firefly luciferase reporter vector (30).

## SUPPLEMENTARY DATA

Supplementary Data are available at NAR Online: Supplementary Table 1, Supplementary Figures 1–4 and Supplementary Methods.

## FUNDING

All funding for the construction of PromoterCAD was supplied by RIKEN and the National Bioscience Database Center (NBDC) of Japan Science and Technology Agency (JST). R.S.C. is a RIKEN Foreign Postdoctoral Researcher. Funding for open access charge: RIKEN.

*Conflict of interest statement.* None declared.
